# Can Peripheral Blood-Derived Gene Expressions Characterize Individuals at Ultra-high Risk for Psychosis?

**DOI:** 10.1162/CPSY_a_00007

**Published:** 2017-12-01

**Authors:** Wilson Wen Bin Goh, Judy Chia-Ghee Sng, Jie Yin Yee, Yuen Mei See, Tih-Shih Lee, Limsoon Wong, Jimmy Lee

**Affiliations:** 1 School of Biological Sciences, Nanyang Technological University, Singapore; 2 Department of Computer Science, National University of Singapore, Singapore; 3 Department of Pharmacology, Yong Loo Lin School of Medicine, National University of Singapore, Singapore; 4 Research Division, Institute of Mental Health, Singapore; 5 Neuroscience and Behavioral Disorders Program, Duke–National University of Singapore Medical School, Singapore; 6 Department of Pathology, Yong Loo Lin School of Medicine, National University of Singapore, Singapore; 7 Lee Kong Chian School of Medicine, Nanyang Technological University, Singapore

**Keywords:** ultra-high risk, psychosis, feature selection, gene expression

## Abstract

The ultra-high risk (UHR) state was originally conceived to identify individuals at imminent risk of developing psychosis. Although recent studies have suggested that most individuals designated UHR do not, they constitute a distinctive group, exhibiting cognitive and functional impairments alongside multiple psychiatric morbidities. UHR characterization using molecular markers may improve understanding, provide novel insight into pathophysiology, and perhaps improve psychosis prediction reliability. Whole-blood gene expressions from 56 UHR subjects and 28 healthy controls are checked for existence of a consistent gene expression profile (signature) underlying UHR, across a variety of normalization and heterogeneity-removal techniques, including simple log-conversion, quantile normalization, gene fuzzy scoring (GFS), and surrogate variable analysis. During functional analysis, consistent and reproducible identification of important genes depends largely on how data are normalized. Normalization techniques that address sample heterogeneity are superior. The best performer, the unsupervised GFS, produced a strong and concise 12-gene signature, enriched for psychosis-associated genes. Importantly, when applied on random subsets of data, classifiers built with GFS are “meaningful” in the sense that the classifier models built using genes selected after other forms of normalization do not outperform random ones, but GFS-derived classifiers do. Data normalization can present highly disparate interpretations on biological data. Comparative analysis has shown that GFS is efficient at preserving signals while eliminating noise. Using this, we demonstrate confidently that the UHR designation is well correlated with a distinct blood-based gene signature.

## INTRODUCTION

Prodromal (early) intervention has reportedly beneficial effects in attenuating, delaying, and even preventing psychosis onset (McGlashan et al., 2003; McGorry et al., 2002; Morrison et al., 2004). Thus high-risk individuals yet to develop full-blown psychosis must be identified. Without genetic evidence, positive identification of ultra-high-risk (UHR) individuals is achieved via interview-based tests: the Comprehensive Assessment of at Risk Mental State (CAARMS; Yung et al., 2002) and the Structured Interview of Prodromal Syndrome (McGlashan, Miller, & Woods, 2001). Both assess risk via a panel of scored clinical traits, including the intensity, frequency, and duration of psychosis symptoms and risk factors (e.g., family history).

UHR designation is useful: Approximately 10%–50% of positively identified individuals convert to psychosis within a year (Haroun, Dunn, Haroun, & Cadenhead, 2006; Lencz, Smith, Auther, Correll, & Cornblatt, 2003; Mason et al., 2004; Yung et al., 2006). But these phenotype-based tests are subjective, and notably, UHR designation has low precision: 50%–90% of UHRs do not convert within a year. Thus prodromal detection requires improvement, and we must leverage objective data to reveal pathophysiology and perform risk assessment.

We look to high-throughput gene measurements (genomics) as objective data. Earlier studies reported identifiable gene expressional differences between psychotic and healthy individuals in both blood and brain tissues (Bowden et al., 2006; Maycox et al., 2009; Vawter et al., 2004). But while we may observe statistically significant expressional differences, whether these signatures have diagnostic value is subjective. Furthermore, statistical feature selection is complex, and different normalization approaches, statistical thresholds, and multiple-test corrections can produce highly varied outcomes (Goh & Wong, 2016a). Suppose hundreds of genes are potential candidates: Naive reliance on and ranking of significance based on *p* values (Wang, Sue, & Goh, 2016) do not account for autocorrelations among genes (non independence), sampling bias and phenotypic relevance. Moreover, hypothesis-based statistical testing is commonly misinterpreted: When the null hypothesis is rejected in favor of the alternative because of insufficient evidence, it does not make the alternative statement (there is a difference) automatically true (Venet, Dumont, & Detours, 2011). Where many variables are correlated (but phenotypically irrelevant), any random selection of genes can have equal if not better predictive power. This implies, across different studies, that identified signatures will vary. Indeed, attempts toward biomarker development for UHR differentiation (Lee et al., 2012; Takahashi et al., 2010) have been met with skepticism, as noticeably, signatures identified in one study are irreproducible in another (Bray, 2008; Iwamoto & Kato, 2006). Nonetheless, we assert that careful experimental design and fair-handed analysis can yield reproducible and useful functional insights (Wang, Sue, & Goh, 2016).

Brain tissue is ideal for inferring prodromal psychosis, but obviously, invasive surgical procedures are not feasible for routine diagnosis. Easily extracted surrogate tissues pos ing minimal risks to subjects are preferable but discernably less reliable. Blood is a convenient surrogate, but more importantly, blood has been reported to be viable for providing a “neurological footprint” (Cai et al., 2010). Some studies have supported strong blood–brain gene correlations at the gene level (e.g., Sullivan, Fan, & Perou, 2006), while others have presented a less certain view: Blood–brain gene correlations are not conserved at the gene level but rather at the modular (subnetworks and pathways) level (e.g., Hess et al., 2016). Indeed, differential genes observed in blood are influenced by input from many other tissues. Given careful subject selection, background elimination, and reproducibility checks, it is possible to obtain a signal consistent with changes in the brain’s pathophysiological status (Jasinska et al., 2009).

Using blood-based genotyping, our first aim is to determine whether a consistent gene expression signal differentiating UHR and non-UHR groups exists. The second aim, a prelude to reproducible statistical feature selection, is to evaluate the impact of various normalization techniques. Third, we evaluate the reproducibility and relevance of gene signatures.

## METHODS AND MATERIALS

### Study Design

Participants are drawn from the Longitudinal Youth-at-Risk Study (LYRIKS), a prospective observational study on youths susceptible to psychosis (Lee et al., 2013). LYRIKS participants are drawn from mental health care and community-based services and from various educational institutions in Singapore. Eligibility requirements mandate a narrow age range (14–29 years), no existing antipsychotic treatment, no other psychotic disorder or neurological disease, and no illicit substance use history. UHR status is ascertained using CAARMS, and positive identification is made if subjects exhibit attenuated psychotic symptoms (APS), brief limited intermittent psychotic symptoms (BLIPS), or general vulnerability (Yung et al., 2002). Healthy controls in this study are participants who did not fulfill UHR criteria and had no psychiatric disorders when evaluated on the Structured Clinical Interview for DSM–IV Axis I Disorders (First, Spitzer, Gibbon, & Williams, 1996). Details on LYRIKS’s study methodology are obtainable from prior publications (Lee et al., 2013; Mitter, Nah, Bong, Lee, & Chong, 2014). The dataset comprises 55 UHR subjects and 28 healthy controls (Table 1). Gender and ethnic ratios are balanced between subjects (UHRs) and healthy controls (non-UHRs) to minimize factor proportion-imbalance disparities (Patil, Bachant-Winner, Haibe-Kains, & Leek, 2015).

**Table 1.** Study design and factor description of study sample

**Status**	***N***	**Subgroups**	**Mean age (years)**	**Gender, *N* (%)**	**Ethnicity, *N*** **(%)**
UHR subjects	56	APS: 43 (76.8%)	22.1	Male: 21 (75.0%)	Chinese: 21 (75.0%)
		BLIPS: 3 (5.4%)		Female: 7 (25.0%)	Malay: 7 (25.0%)
		Vulnerable: 15 (26.8%)			
Healthy controls	28	None	22.5	Male: 21 (75.0%)	Chinese: 21 (75.0%)
				Female: 7 (25.0%)	Malay: 7 (25.0%)

### Gene Expression Measurement

Peripheral blood is drawn immediately following assessment into a Tempus Blood RNA tube (Applied Biosystems, Foster City, CA) and is stored at − 80°C until RNA extraction. Total blood RNA is extracted using a Tempus Spin RNA isolation kit (Applied Biosystems) and amplified using an Illumina TotalPrep RNA amplification kit (Ambion, Austin, TX). mRNA expression profiles are assessed on Illumina HumanHT-12 v4 Expression BeadChip arrays. Experimental quality controls are performed prior to RNA amplification and before RNA hybridization to ensure that RNA concentrations are ≥100 ng/ml, A_260_/A_280_≥2.0, and clearly defined ribosomal peaks were seen on agarose gel. All procedures adhered to design protocols as per manufacturer recommendations. The readings of the beads in the array are analyzed by Illumina iScan following hybridization.

### Microarray Quality Control

Probe intensities on the Illumina Microarray BeadChips are summarized using GenomeStudio (Illumina, San Diego, CA). To ensure read quality, sample-based and probe-based quality control (QC) is performed. In sample QC, samples with signal-to-noise ratio below 10 or samples with intensity of negative control probes higher than control probes are considered unreliable and discarded. In probe QC, probes with detection *p* values below 0.05 or below background are considered undetectable and discarded. A total of 84 subjects (56 UHRs + 28 non-UHRs) across 18,029 gene probes meets QC standards.

### Normalization Methods

Following background subtraction, probe-to-gene name mapping, and following median centering of expression, four normalization approaches are applied on the quantified data: Log-conversion (None), quantile normalization (Quantile), gene fuzzy scoring (GFS; Belorkar & Wong, 2016), and surrogate variable analysis (SVA; Leek & Storey, 2007).

Strictly speaking, None is not true normalization; we use this primarily as a negative control and to facilitate parametric statistics. We use the natural log, such that for each observed measurement *x*, the transformed value is log_*e*_ *x*, where *e* = 2.718. None reduces the effect of high orders of magnitude (because *e* ∼ 3, the data range is reduced by three orders) and improves the distribution symmetry. However, None is usually insufficient to render samples cross comparable and is usually accompanied by a second data transformation, typically *z* scaling, linear interpolation, or quantile normalization.

Quantile is a gold-standard data transformation procedure and reportedly performs well in stabilizing variance in Illumina BeadChip HT-12 (Schmid et al., 2010). Although we could include linear interpolation and *z* scaling, these are already known to be inferior to Quantile anyway and detract from more interesting new methods that explicitly deal with heterogeneity. GFS is an unsupervised signal-boosting transformation (Belorkar & Wong, 2016; Wang et al., 2016). In GFS, the log-transformed expression matrix is transformed by weighting individual genes per sample based on expression ranks. GFS uses two thresholds, θ_1_ and θ_2_. Features with ranks above θ_1_ are assigned a weight of 1, features with ranks between θ_1_ and θ_2_ are assigned an interpolated weight between 1 and 0, and features with ranks below θ_2_ are weighted as 0. Let *r*(*g*_*i*_,*p*_*j*_) be the rank of a biological feature *g*_*i*_ in patient *p*_*j*_ and *q*(*p*_*j*_,θ) be the rank corresponding to the upper θth level of feature ranks in *p*_*j*_. The GFS score *s*(*g*_*i*_,*p*_*j*_) assigned to feature *g*_*i*_ for patient *p*_*j*_ is determined by the function

$$s\text{(}g_{i},p_{j}\text{)}=\left\{\begin{array}{c} 1\\ \frac{r\text{(}g_{i},p_{j}\text{)}-q\text{(}p_{j}{,\theta }_{2}\text{)}}{q\text{(}p_{j}{,\theta }_{1}\text{)}-q\text{(}p_{j}{,\theta }_{2}\text{)}}\\ 0 \end{array}\right.\begin{array}{c} \;\text{if}\;q\text{(}p_{j}{,\theta }_{1}\text{)}<r\text{(}g_{i},p_{j}\text{)},\\ \;\text{if}\;q\text{(}p_{j}{,\theta }_{2}\text{)}>r\text{(}g_{i},p_{j}\text{)}\geq q\text{(}p_{j}{,\theta }_{2}\text{)},\\ \;\text{otherwise}. \end{array}$$

As arbitrarily defined thresholds, θ_1_ and θ_2_ can take on different values, for example, the default settings in GFS set θ_1_ to 5% and θ_2_ to 15%. However, in their evaluations, Belorkar and Wong (2016) stated that varying θ_1_ between 5% and 10% and θ_2_ between 15% and 20% produces similar results. Comparing GFS against standard normalization techniques—for example, mean-scaling, *z*, and quantile normalization—GFS consistently gives better class discrimination, is robust against batch effects, provides improved power even when sampling at small sample sizes, and facilitates reproducible selection of biologically relevant features.

SVA is applied on the log-transformed expression matrix. In contrast to GFS, SVA is a supervised method—that is, it requires specification of class labels, UHR subject and UHR control, a priori, meaning that the algorithm is aware of class information—and is therefore autobiased toward class effects (Leek & Storey, 2007). It assumes that consistent sources of variation nonassociated with the class factor are likely associated with some unknown heterogeneity factor. Having identified the part of the data not associated with class variation, it then estimates heterogeneity factors via singular value decomposition. These irrelevant variations (expressed as surrogate variables) are then removed via regression. The beauty of SVA is that we may isolate UHR-specific signals against a backdrop of various confounding factors that we cannot possibly control for, especially genetic factors. Although it is extremely powerful, SVA can be complex to use (Jaffe et al., 2015).

### Principal Component Analysis (PCA)

PCA is a linear summarization technique that collapses high-dimensional data (e.g., thousands of genes) into a lower number of orthogonal dimensions, or principal components (PCs; Giuliani, 2017). We use PCA to observe whether individual samples group by class consistently, without feature selection and regardless of normalization method.

We also use the PCA-generated loading matrix to determine the association of individual PCs against each factor (class, gender, and ethnicity). Assuming that a factor may have more than two levels, association is evaluated using the nonparametric Kruskal–Wallis (KW) test (*p* ≤ 0.05).

### Feature Selection and Multiple-Test Correction

The *F* test is used to evaluate a differential expression for each gene, following Benjamini–Hochberg (BH) multiple-test correction.

The *F* statistic is expressed as

$$F=\frac{\sum_{i=1}^{K}n_{i}\frac{{\left({\bar{Y}}_{i}-\bar{Y}\right)}^{2}}{K-1}}{\sum_{i=1}^{K}\sum_{j=1}^{n_{i}}\frac{{\left(Y_{i,j}-{\bar{Y}}_{i}\right)}^{2}}{N-K}},$$

where *Y*_*i*,*j*_ is the *j*th observation in the *i*th group over *K* groups. *N* is total sample size, and *n*_*i*_ is the size of the *i*th group. $\bar{Y_{i}}$ is the mean within group *i*, and $\bar{Y}$ is the overall total sample mean. *F* is the ratio of intergroup over intragroup variances and is large if the groups are strongly different. The *p* value is determined against the nominal *F* distribution with (*K* − 1, *N* − *K*) degrees of freedom.

### Classifier Training and Cross-Validation

#### Random splits + no feature selection

Samples are evenly split into training and validation sets. All features are used to train a shrunken-centroid classifier (Dabney, 2005). Cross-validation accuracy is the fraction of correctly predicted class labels (UHR subject and UHR control) in the validation set. This is repeated 1,000 times for each normalization method.

#### Random splits + feature selection

The same procedure was performed as described earlier, except feature selection in the training set (signature) is performed using *F* test/BH correction (*p* ≤ 0.01).

To demonstrate robustness, given each split, an equal number of random features was selected, and the cross-validation accuracy based on randomly selected features (equal to signature size) was determined.

### Functional Analysis Based on Networks

GeneMANIA (http://www.genemania.org/) is used to evaluate functional relationships among differential genes (Montojo, Zuberi, Rodriguez, Bader, & Morris, 2014). It features an adaptive weighting method to combine various network data, such that after the networks are combined, the differential genes interact maximally with each other, while interacting as little as possible with genes not in the list. This allows GeneMANIA to learn which networks mediate the underlying functional relationship among the differential genes but also permits the inclusion of additional genes strongly associated with the differential set.

Enriched gene ontology annotations associated with the finalized network are also reported, and we used these for checking whether the associated network, induced by the set of differential genes, has strong neurological associations.

## RESULTS

### Ultra-high Risk (UHR) Blood-Derived Gene Expressions Are Strongly Distinct Between Data Normalization Techniques

**Figure 1.**
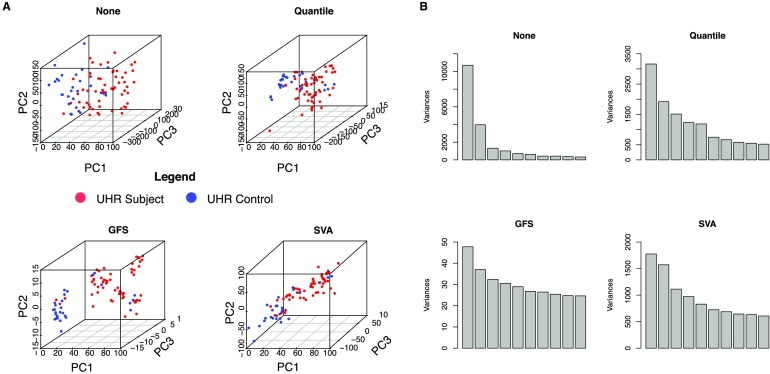
**Preliminary variance-based analysis.** A) PCA scatterplots demonstrating that data normalization can improve the signal-to-noise ratio, enhancing discrimination between sample classes. Note that no feature selection is done here. Here we compare None, Quantile, GFS, and SVA. GFS and SVA seem to boost the class discrimination signal the most. B) Distribution of variance at each PC level shown as a series of bar plots, where the first bar corresponds to PC1, the second corresponds to PC2, and so on. In “None,” note that without any form of normalization, most variance is concentrated in PC1. A high concentration of variance in the first PC is usually indicative of the presence of a large amount of technical artifact. All normalization methods appear to balance the distribution of variance among the subsequent PCs, but also note that the relative scale of remaining variance after GFS and SVA processing is much lesser than for log-converted data.

Previously, it was unknown whether UHR subjects possessed distinct but conserved gene expression profiles. Plotting individual samples using the first 3 PCs without prior feature selection suggests that they do (Figure 1A).

The existence of strong class-distinguishing signals without a priori feature selection is good evidence that UHRs form a distinct class. However, this may be a consequence of data-processing bias. Therefore we rechecked using various data-processing and normalization techniques: None, Quantile, GFS, and SVA. The first two techniques are common, GFS is a signal-boosting transformation, and SVA deals with unwanted heterogeneity.

While the scatterplots present a consistent view that UHR subjects are distinct (Figure 1A), inter- and intracluster variances differ. In None, interclass variability is lower relative to intraclass variability. Quantile, GFS, and SVA all produce significant improvement, demonstrating the importance of normalization. Note that good class separation is expected in SVA anyway.

PCs are high-dimensional projections based on the expression patterns across thousands of genes such that each holds information regarding a conserved pattern of expressional change correlated with various factors (e.g., class, age, gender, ethnicity, and even biological pathways; Giuliani, 2017; Goh & Wong, 2016c). Each gene-based variable loads differently onto each PC, and its loading can be determined from the PCA loading matrix.

We may also investigate PCs for evidence of bias (Figure 1B): A disproportionate proportion of variance in PC1 suggests technical bias, for example, batch effect (Giuliani, 2017; Goh & Wong, 2016c) or spurious correlations (Giuliani, 2017; Goh & Wong, 2016c). Expectedly, None holds a high proportion of total variance in PC1, while total variance is distributed more evenly in the other normalization methods.

The intersample distances change as more PCs are considered, and there is no reason why subsequent PCs (after PC3) should be ignored. Each PC uniquely encapsulates a distinct portion of total variance and is mutually orthogonal. We may deploy the PCs more effectively by quantifying the association of each PC against known factors (class, gender, ethnicity). If sample class is associated significantly among the top PCs, then this supports the scatterplot analysis. Conversely, we may also reverse engineer interesting PCs and identify which genes load strongly onto it (Goh & Wong, 2016c).

To test association, the KW test is used, accounting for scenarios where a factor has more than two levels. If a PC exhibits strong differential behavior between factor levels (*p* ≤ 0.05), then we assume that this factor can explain this PC. As a rule, for good normalization methods, the top PCs should be significantly associated with class, followed by other important factors.

Table 2 is consistent with the scatterplots. To improve visual impact, the KW *p* values are summarized to two decimal places, and boldface indicates a significance level below 0.05. It should be noted that in hypothesis-based statistical testing, the magnitude of the *p* value is actually inconsequential; of importance is only whether it falls below the threshold (Goodman, 1992). Although all PCs can be tested, the top 10 are adequate to make our point regarding factor rankings.

**Table 2.** Significant association between data factors (class, gender, and ethnicity) against each principal component 110

**PC**	**Class**			**Indeterminate Gender**				**Indeterminate Ethnicity**				
	**None**	**Quantile**	**GFS**	**SVA**	**None**	**Quantile**	**GFS**	**SVA**	**None**	**Quantile**	**GFS**	**SVA**
1	**0.00**	0.38	**0.00**	0.38	0.36	**0.02**	0.56	0.34	0.35	**0.01**	0.91	0.97
2	0.16	0.14	**0.00**	**0.00**	**0.01**	0.81	0.85	0.20	0.12	0.69	0.69	0.85
3	0.23	0.05	0.10	0.22	0.25	0.09	**0.01**	0.85	**0.05**	0.95	**0.01**	0.37
4	0.24	**0.00**	0.21	0.73	0.24	0.19	0.36	0.20	0.76	0.86	0.66	0.82
5	**0.00**	**0.00**	0.13	0.21	0.09	0.78	0.16	**0.03**	0.92	0.91	0.95	0.23
6	0.70	**0.02**	0.14	**0.03**	0.25	0.72	1.00	**0.00**	0.95	0.30	0.37	0.64
7	**0.03**	0.27	0.33	0.06	0.87	0.59	0.07	0.30	0.07	0.79	0.11	0.20
8	0.12	**0.02**	0.98	0.14	0.34	0.19	0.94	0.36	0.33	0.62	0.31	**0.01**
9	0.30	0.22	0.08	0.87	0.23	**0.00**	0.22	**0.01**	0.77	0.88	0.90	0.13
10	0.59	0.23	0.86	0.79	**0.01**	0.42	0.74	0.31	0.59	0.65	**0.05**	0.50

*Note*. Boldface indicates significance below 0.05.

Class effect ranks highly. Interestingly, in Quantile, the top two PCs correlate strongly with gender and ethnicity, while class is relegated to lower PCs. This does not mean that a class effect is absent in the first two PCs; rather, these PCs exhibit a stronger association with other factors. GFS brings class, gender, and ethnicity to the fore (PCs 1–3). Note that GFS is unsupervised and robust against heterogeneity and technical bias (Belorkar & Wong, 2016; Wang et al., 2016), so this is strong evidence that UHRs are genotypically distinct.

In SVA, various factors besides class can be considered. Because we only supplied class, ethnicity and gender effects are suppressed. It is known that psychosis is subclassifiable via these factors (Bresnahan et al., 2007; Canuso & Pandina, 2007). Our test results agree with expectations: Class association ranks highly in SVA (PC2), while gender and ethnicity are relegated to PCs 8 and 9, respectively.

In None, Quantile, and GFS, UHR subjects and controls are consistently separated (Figure 1), although intra- and intercluster distances vary. PC-based factor association suggests that class ranks highly. The unsupervised approach of GFS greatly improves signal-to-noise ratio, and relevant factors rank highly among the top PCs following GFS transformation.

### Statistical Feature Selection Depends Strongly on the Data Normalization Technique

We expect statistical feature selection to provide disparate information regarding differential genes (Figure 1A). Biological relevance is inferred from statistical testing (differential expression), but there are many caveats. While different statistical feature-selection methods can yield different results (Christin et al., 2013; Langley & Mayr, 2015), normalization is also important—and less well explored.

If normalization has limited impact on feature selection, then given a single feature-selection method, selected features from differently normalized datasets should be similar. Genes are selected using the *F* test/BH correction (*p* ≤ 0.01). The *F* test is used to ensure cross-comparability of the other methods with SVA as it reduces overall variability, while the degrees of freedom, given any statistical test, remain the same. While this amplifies effect size (such that if a gene is truly differential, it will be more easily detected), it also increases the false-positive rate (a nondifferential gene is also likely to be reported as significant). Thus SVA requires an appropriate correction (provided within the SVA package on the *F* test).

**Figure 2.**
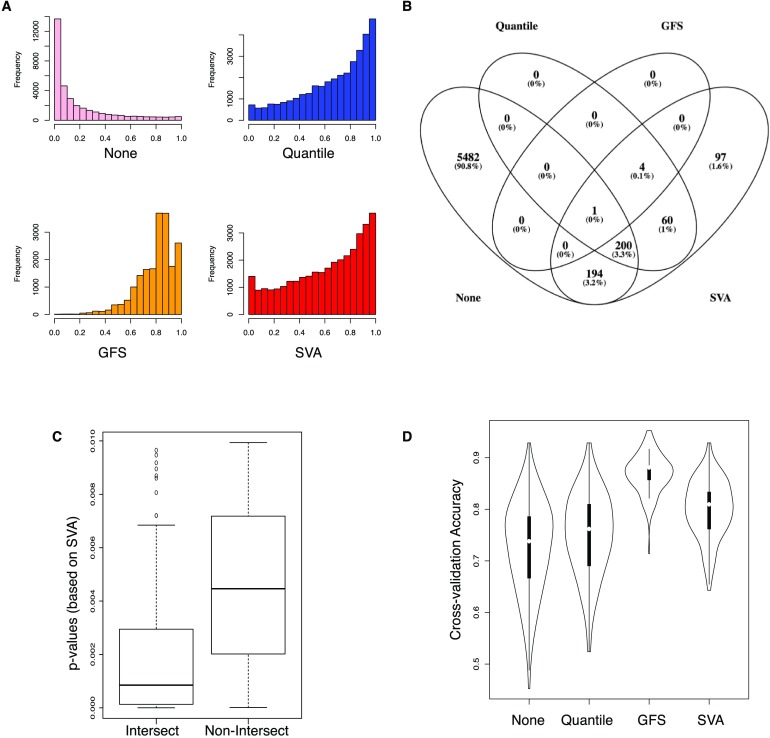
**How normalization affects statistical feature selection and prediction modeling.** A) Histograms showing the *p* value distributions (*x* axis) following feature selection (based on the *F* test) and corrected for multiple testing via BH. Data are processed in four ways (None, Quantile, GFS, and SVA). The importance of normalization is obvious here. With simple log-conversion, most gene features will be reported as significant, and we should expect that many of these will be false positives. The *p* value distributions for Quantile and SVA are more within expectations, while GFS tends to be highly conservative here. B) Significant feature overlap based on a cutoff of 0.01. None, Quantile, GFS, and SVA report a total of 5,877, 256, 5, and 556 significant genes, respectively. Among these, only one gene (*MAGEB16*) is common among all four methods. The overlaps with GFS tend to be deeper with Quantile and SVA. C) Distributions of *p* values (based on SVA’s set of *p* values following *F* test and BH correction) showing that intersecting genes (common between Quantile, GFS, and SVA) are more significant than those that are not common among them. We disregarded the 5,482 significant genes in None, as they are quite likely to be false positives anyway. D) Cross-validation tests demonstrating that GFS, followed by SVA, tends to pick more relevant genes and build better models using the shrunken-centroid classifier. Data are evenly split into training and validation sets. All features were used to train the classifier. Cross-validation accuracy is the total number of correctly predicted class labels (control and subject) in the validation dataset (where 0 means no class labels were correctly predicted and 1 means all were correctly predicted). This is repeated 1,000 times to generate the violin plot, as shown.

Based on *p* value distributions (extreme right skew), it is clear that None is problematic (Figure 2A). A standard cutoff at 0.05 introduces many statistically significant features. After raising the cutoff to 0.01, ∼6,000 genes are still declared differential (likely most are false positives). Note that the issue is unresolvable via *p* value ranking (and selecting the top *n* genes), because rank instability increases as *p* values get smaller (Wang et al., 2016).

The other methods’ *p* value distributions are more reasonable, suggesting that only a few genes are differential. Quantile completely reverses the *p* value distribution (Figure 2A). GFS is extremely stringent, while SVA predicts slightly more features than Quantile.

None, Quantile, GFS, and SVA select a total of 5,877, 256, 5, and 556 significant genes, respectively (*p* ≤ 0.01; Figure 2B). Among these, only one gene (*MAGEB16*) is shared. Quantile, GFS, and SVA exhibit deeper overlaps with each other. In particular, GFS shares 80% (four out of five) of its selected features with Quantile and SVA, but not with None. And these overlapping genes are important: Their associated *p* values (based on SVA) are far lower (and therefore more significant) than nonoverlapping genes (Figure 2C). Thus we conclude that normalization has strong downstream implications for feature selection.

We are concerned with which approach produces cleaner data. If noise were eradicated, then even without feature selection, the best approach would produce highly accurate models anyway. To do this, we turn to cross-validation without feature selection. Both GFS and SVA reduce heterogeneity, particularly for GFS where the median cross-validation accuracy is highest at 88% (Figure 2D). This confirms that GFS and SVA remove unwanted confounding variation, unlike None and Quantile. Therefore we conclude that GFS is the best normalization technique and is more likely to provide a useful signature.

### Gene Fuzzy Scoring Gene Signature Is Functionally Relevant and Beats Randomly Generated Signatures

GFS selected five genes (*IGSF1*, *LOC653712*, *LRRTM2*, *MAGEB16*, and *PCSK1*; *p* ≤ 0.01). The stringent cutoff is used for limiting the high number of selected features due to log-conversion. For phenotypic correlation, we may bolster sensitivity by relaxing the *p* value cutoff to 0.05 (additional genes are *CDH11*, *CXORF55*, *CYR61*, *NOVA2*, *NTRK2*, *PARVA*, and *RBMY1J*).

**Figure 3.**
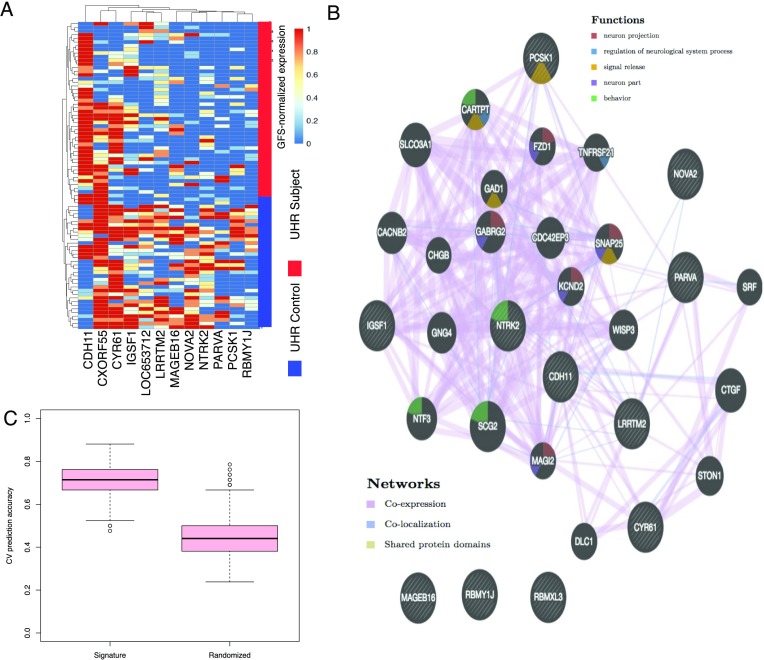
**Gene fuzzy scoringbased gene signature is functionally relevant.** A) An unsupervised clustering method (hierarchical clustering; Euclidean distance and average linkage) on the set of GFS significant features (the ones in bold are the original five at a cutoff of 0.01, while the additional seven are included based on a cutoff of 0.05), yielding good separation between our sample classes. The cutoff was loosened to 0.05 to include more genes and boost sensitivity in functional analysis. B) Functional network (derived from GeneMANIA) among the significant GFS genes, pointing toward neurological functions and a high level of interconnectivity among other undetected genes. Despite its strong presence as a significant feature, *MAGEB16* does not appear to be functionally associated with the other genes. C) Half samples used for training following statistical feature selection (signature), the remaining half for validation. The cross-validation prediction accuracy is the proportion of correctly predicted validation class labels. In each round, a random signature equal to the size of the inferred signature is also generated, and its cross-validation performance is evaluated similarly. Although classifier accuracy fell for GFS (compare Figure 2D), it strongly outperforms random signatures, suggesting that signatures inferred from GFS are more likely meaningful or relevant. This is not so for other normalization methods (compare Goh et al., 2017, Supplementary Figure 1).

We use the term *signature* liberally: We do not mean it in the sense that it has valid diagnostic capabilities in the greater UHR population but rather use it to indicate a set of genes being evaluated for class relevance and predictive power, within the confines of our study design. Although small, the GFS signature can distinguish UHR subjects and controls (hierarchical clustering; Euclidean distance; Ward’s linkage; Figure 3A); that is, we may replicate the strong PC-based interclass segregation (Figure 1A) with only 12 genes.

To determine functional interrelationships, we supplied the GFS signature to GeneMANIA (Montojo et al., 2014; Vlasblom et al., 2015) and searched for functional links based on coexpression, co-localization, and shared functional domains (Figure 3B). We also recovered additional implicated genes (in gray) strongly associated with the signature. The induced gene network (including both signature and implicated genes) is strongly associated with neurological functions, including genes associated with behavior, signal release, and neuron projection.

GFS provides the highest cross-validation accuracies (Figure 2D), suggesting that it removed a high amount of noise. Not all genes are useful though; we may simplify model building and explanation if we isolate only differential genes (via statistical feature selection) to form signatures. Unfortunately, this is not straightforward. Venet et al. (2011) demonstrated that inferred signatures across breast cancer studies seldom generalize and do not outperform random signatures. In fact, the larger the gene signature is, the more likely it is that randomized gene sets will outperform it. Although the breast cancer signatures in themselves are predictive, they offer no more information than any random assortment of genes, meaning that they cannot be used to reveal mechanism or biological insight. This occurs because a large fraction of genes are autocorrelated with cancer but play no role in disease progression. It is unclear if this is also true for UHR.

We repeated cross-validation with feature selection using the *F* test/BH correction (*p* ≤ 0.01). It is interesting that feature selection with GFS reduces classifier accuracy (Figure 3C). This means that although the top five GFS genes have some predictive power, UHRs are quite heterogeneous. Moreover, despite the drop in classifier accuracy, GFS signatures do much better than random signatures (Figure 3C).

Feature selection is important. Certainly the *F* test failed to preserve the original high accuracy given all features. However, we also removed much noise, such that randomly picked gene signatures do no better. In comparison, feature selection with Quantile, SVA, and None appears to preserve, or improve, classifier accuracy, but the random signatures accompanying these also do well (Goh et al., 2017, Supplementary Figure 1). Inferred signatures derived from these normalization methods are likely less meaningful.

It is worthwhile to evaluate feature-selection stability during cross-validation. Goh et al. (2017, Supplementary Figure 2A; compare Figure 2D) suggested that None/SVA/Quantile-normalized data still contain some noise and thus that feature selection helps a little, whereas the GFS-normalized data have less noise, so feature selection does not help as much. Moreover, the feature-selection stability across all normalization is poor, although GFS is still superior in this respect (Goh et al., 2017, Supplementary Figure 2B). Moreover, the gene reproducibility index (see Goh et al., 2017, Supplementary Methods) for GFS is the highest and approximately five times more reproducible than SVA (Goh et al., 2017, Supplementary Figure 2B).

## DISCUSSION

### Limitations of the Current Study and Follow-Up Investigations

The present study demonstrates that UHR subjects possesses distinct blood-based signatures, but we do not claim generalizability. Ostensibly, the sample size is limited. However, given careful subject selection to facilitate cross-comparability and minimize background (Qin et al., 2014), UHR subjects can be consistently distinguished from UHR controls, but normalization can present disparate views on the underlying biology.

Good normalization methods should minimize or eliminate technical bias and increase signal-to-noise ratio (i.e., amplify signal, reduce noise). We should not rely on feature-selection methods to work properly without proper data cleaning. As it turns out, most feature-selection methods are not resistant to technical bias or noise (Goh & Wong, 2016c).

Commonly used normalization methods fall short, while newer approaches, designed for dealing with heterogeneity, are superior. The unsupervised approach of GFS is promising. In follow-up studies, we aim to determine if GFS signatures are generalizable in larger cohorts and across a wide variety of independent studies on the same phenotype.

This is the first study to investigate a genetic basis underlying UHRs. We are positive that specific blood-based gene expression signatures from UHR subjects can be elucidated, which can in turn facilitate accurate early detection and therapeutic intervention.

We are also aware that issues pertaining to blood–brain gene expression correlations are not addressed (Cai et al., 2010; Jasinska et al., 2009). We avoid overinterpretation of mechanism but merely point out that the GFS blood signature is phenotypically coherent. In subsequent studies, we aim to relate blood- and brain-derived UHR gene signatures.

Although we focus mostly on the downstream effects of normalization in functional analysis, we also introduce some useful analytical techniques. PCA and normalization are discussed in subsequent sections. Here we comment on our cross-validation setup where we randomly sampled from UHR subject and control, performed a standard feature selection, trained a classifier, and obtained a cross-validation accuracy. This is standard procedure, but it does not tell us whether this accuracy is meaningful in that the selected features are phenotypically relevant in an exclusive sense (Goh & Wong, 2016b). This can be counterchecked by randomly sampling an equal number of features and checking if the derived cross-validation accuracies do better. An empirical *p* value for the cross-validation accuracy is the number of times a random signature does better than the observed. This is a powerful approach, because it deals specifically with the issue of random signature superiority (Venet et al., 2011): If the signature is relevant, it must be exclusive, and it must perform better than any random assortment of genes. Intuitively, a signature passing this check is more likely to be generalizable.

Finally, when study designs are imbalanced, it is important never to derive statistics for each gene across samples, as this would lead to test-set bias during classifier building (Patil et al., 2015). Test-set bias occurs when the classification of any subject or tissue in a dataset is dependent on the composition of the other subjects or tissues as the expression of each protein or gene is normalized with respect to the entire dataset (Patil et al., 2015). Any gene signatures derived based on any normalization strategy that has dependency across subjects or tissues of the test set will be irreproducible when the population changes composition or size. Thus readers should be careful when encountering any normalization method that adjusts across multiple subjects or tissues rather than information strictly within a subject or tissue itself (Goh & Wong, 2016a). The problem is easily avoided simply by not normalizing across samples but rather within samples, as we have done here. However, the unsolved problem is that we have less information regarding variance in UHR controls, and this can indeed undermine our analysis methods. But as mentioned earlier, we do not claim generalizability but work within the confines of this study.

### Normalization Is Vital and Contributes Toward Reproducible Signatures

It is clear that log-conversion (None) alone is insufficient and may produce false effects. The oft-used quantile normalization (Quantile) provides considerable improvement but may not be good enough for practical applications. Finally, the highly sophisticated technical variability removal method, SVA, is powerful, but as a supervised method, it is susceptible to the veracity of the class labels: If wrong, for example, owing to misdiagnosis, then SVA can make mistakes. Finally, we introduce the unsupervised signal-boosting transformation GFS, which provides the highest data-cleaning utility (Figure 2D).

Although GFS’s performance suffers when combined with a feature-selection approach, it is the only method that can strongly outperform random signatures (Figure 3C; Goh et al., 2017, Supplementary Figure 1). This is vital, because when applied on random subsets of data, classifiers built with GFS are “meaningful.” If classifiers built with random genes are accurate as well, this means that many genes in the dataset are noncausally correlated with the phenotype, such that any inferred signature has no real meaning anyway. Although the other approaches generally produce higher classifier accuracies following feature selection, only the GFS-derived classifiers outperform the randomly built classifiers. This provides greater confidence that the five-gene GFS signature inferred from the full dataset is not associated with the UHR phenotype simply by chance.

### PCA Can Be Used More Effectively

PCA scatterplots are commonly used for determining the presence of batch effects and checking sample separation before and after feature selection. Typically, analysis is limited to PCs 1–3, with the assumption that most variance is contained therein (the remaining variance being uninteresting). This is not always the case where, following certain normalization approaches, the distribution of variance among PCs becomes more balanced (Figure 1B). Although each subsequent PC contains a lower proportion of total variance, it is not negligible, and there is no valid reason to discard any of them. Conversely, a high proportion of variance in PC1 suggests high technical bias (Goh & Wong, 2016c). It is important to deal with bias, instead of blindly accepting the top three PCs.

We may use PCs for conducting correlation checks with known factors: If technical batches are known, we may determine which PC is associated with batch. If correlated with PC1, batch effect dominates, and skews feature selection. Similarly, examining PC correlations with other factors determines if they contribute strongly and meaningfully to the model. In particular, we expect gender and racial effects to be present. Indeed, these are correlated with top PCs, except in SVA. (This is expected, as we did not supply this to SVA, and gender/racial effects were thus suppressed.)

Detailed PC examination reveals the influence of various factors, but we need to figure out which genes are responsible. This is possible (but we did not deploy this technique explicitly here): Suppose PC1 is strongly associated with batch; we can go back to the PCA loading matrix and isolate genes that load strongly onto PC1, thus isolating the batch effect–susceptible genes (Goh & Wong, 2016c). If PC1 is strongly and uniquely associated with class, then we can extract the “class differential” genes from there. This provides useful information, in addition to statistical feature selection.

Certainly PCA is a very versatile technique, and it can be used more creatively, to powerful effect.

### UHR Phenotype Class Is Inferable via Blood-Based Signatures

Although our sample size is moderate, we are able to consistently observe strong differences between UHRs and non-UHRs, even without feature selection. Examination of the gene signature derived from GFS suggests association with known psychosis genes. Many of these genes are implicated in psychosis and schizophrenia: *PCSK1* is reported to be dysregulated (Hokama et al., 2014). *LRRTM2* is a maternally suppressed gene that is associated paternally with handedness and schizophrenia (Francks et al., 2007). *NTRK2* together with *BDNF* is associated with paranoid schizophrenia (Lin et al., 2013). *PARVA* is associated with cognitive control, the loss of which is a core trait in schizophrenia (Lewis, Curley, Glausier, & Volk, 2012). Although some genes (e.g., *MAGEB16*) are reported consistently, they are immune associated and do not seem consistent with the UHR phenotype. However, the immune system and schizophrenia have a long history of intricate association (Horvath & Mirnics, 2014; Jenkins, 2013; Muller & Schwarz, 2010; Vetlugina, Lobacheva, Semke, Nikitina, & Bokhan, 2016). Although it is useful to be able to phenotypically relate gene signatures, doing so does not offer nonquantifiable evidence. Therefore additional means to demonstrate signature specificity and power are necessary.

Taken together, the results suggest that blood-based diagnosis is useful for characterizing UHRs.

## CONCLUSION

A distinct peripheral blood-based gene expression signature from UHR subjects is identifiable. Although feature-selection approaches are important, data normalization is equally vital, as procedures that deal with heterogeneity can give rise to more stable signatures (following feature selection) with high predictive power.

Given good normalization, and demonstrated independent reproducibility, blood-based gene signatures have the potential to help identify UHRs accurately and, it is hoped, improve clinical outcomes via early intervention.

## AUTHOR CONTRIBUTIONS

W. W. B. Goh and L. S. Wong designed and conducted the bioinformatics analyses. J. C. G. Sng performed the sample preparations. J. Y. Yee and Y. M. See collected patient samples. T. S. Lee and J. Lee profiled the patient cohort.

## FUNDING INFORMATION

The Singapore Translational and Clinical Research in Psychosis (J.Y.Y., Y.M.S., T.S.L., J.L.) is supported by the National Research Foundation Singapore under the National Medical Research Council Translational and Clinical Research Flagship Program (grant NMRC/TCR/003/2008). L. W. is supported in part by a Kwan Im Thong Hood Cho Temple chair professorship.
